# Proteomic Analysis Identifies an NADPH Oxidase 1 (Nox1)-Mediated Role for Actin-Related Protein 2/3 Complex Subunit 2 (ARPC2) in Promoting Smooth Muscle Cell Migration

**DOI:** 10.3390/ijms141020220

**Published:** 2013-10-11

**Authors:** Imad Al Ghouleh, Andrés Rodríguez, Patrick J. Pagano, Gábor Csányi

**Affiliations:** 1 Vascular Medicine Institute, 12^th^ Floor BST, 200 Lothrop Street, University of Pittsburgh, PA 15261, USA; E-Mails: ima6@pitt.edu (I.A.G.); anigrodriguez@gmail.com (A.R.); 2 Department of Pharmacology & Chemical Biology, 13^th^ Floor BST, 200 Lothrop Street, University of Pittsburgh, PA 15261, USA

**Keywords:** vascular smooth muscle cell, migration, NADPH oxidase, oxidative stress, ARPC2

## Abstract

A variety of vascular pathologies, including hypertension, restenosis and atherosclerosis, are characterized by vascular smooth muscle cell (VSMC) hypertrophy and migration. NADPH oxidase 1 (Nox1) plays a pivotal role in these phenotypes via distinct downstream signaling. However, the mediators differentiating these distinct phenotypes and their precise role in vascular disease are still not clear. The present study was designed to identify novel targets of VSMC Nox1 signaling using 2D Differential In-Gel Electrophoresis and Mass Spectrometry (2D-DIGE/MS). VSMC treatment with scrambled (Scrmb) or Nox1 siRNA and incubation with the oxidant hydrogen peroxide (H_2_O_2_; 50 μM, 3 h) followed by 2D-DIGE/MS on cell lysates identified 10 target proteins. Among these proteins, actin-related protein 2/3 complex subunit 2 (ARPC2) with no previous link to Nox isozymes, H_2_O_2_, or other reactive oxygen species (ROS), was identified and postulated to play an intermediary role in VSMC migration. Western blot confirmed that Nox1 mediates H_2_O_2_-induced ARPC2 expression in VSMC. Treatment with a p38 MAPK inhibitor (SB203580) resulted in reduced ARPC2 expression in H_2_O_2_-treated VSMC. Additionally, wound-healing “scratch” assay confirmed that H_2_O_2_ stimulates VSMC migration via Nox1. Importantly, gene silencing of ARPC2 suppressed H_2_O_2_-stimulated VSMC migration. These results demonstrate for the first time that Nox1-mediated VSMC migration involves ARPC2 as a downstream signaling target.

## 1. Introduction

Vessel wall remodeling plays a key role in the development of vascular disorders, including systemic hypertension and atherosclerosis [1,2]. Proliferation and migration of vascular smooth muscle cells (VSMC) as well as extracellular matrix rearrangement are important early mechanisms in the pathogenesis of vascular disease [3]. However, the molecular mechanisms differentiating these events are not well defined.

Reactive oxygen species (ROS), namely superoxide anion (O_2_^•−^) and hydrogen peroxide (H_2_O_2_), mediate signal transduction pathways contributing to the pathophysiological responses of VSMC, including proliferation, migration, and hypertrophy [4,5]. A major source of ROS in VSMC during vascular remodeling is the NADPH oxidase (Nox) family of enzymes [6]. Rodent conduit artery VSMC express Nox1 and Nox4 [7]. Nox1 primarily generates O_2_^•−^, while Nox4 appears to constitutively produce H_2_O_2_[8,9]. In addition, these Nox isoforms serve distinct functions within cells, purportedly a consequence of their distinct intracellular compartmentalization, unique mechanism of regulation and activation, and the ROS they produce. Our laboratory has recently demonstrated that pathophysiologically-relevant concentrations of H_2_O_2_ stimulate Nox1-derived O_2_^•−^ generation in rat aortic VSMC, leading to activation of apoptosis signal-regulating kinase 1 (Ask1). These data delineated a previously unknown molecular cascade by which feed-forward ROS signaling in the vascular wall is propagated [10]. Irrespective of the stimulant, previous data demonstrate that VSMC Nox1 activation is at the core of a network of vascular ROS signaling [10,11]. The means by which Nox1 directs phenotypic changes in the vascular wall is an ongoing area of intense interest in our laboratory.

Previous studies have established that Nox-derived ROS play an important role in VSMC migration [11]. Indeed, conduit artery VSMC overexpressing p22*^phox^*, the membrane-bound subunit associated with the Nox catalytic core, demonstrate increased migratory ability compared to wild type controls [12]. In order to identify novel targets of Nox1 activation in VSMC, the current study employed 2D Differential In-Gel Electrophoresis and mass spectrometry (2D DIGE/MS). Proteomic analysis using 2D DIGE/MS revealed a Nox1-mediated upregulation of VSMC actin-related protein 2/3 complex subunit 2 (ARPC2). The data demonstrated that VSMC migration via Nox1 involves ARPC2, shedding light on a new signaling pathway in vascular biology.

## 2. Results and Discussion

### 2.1. 2D-Gel Proteomic Analysis Identifies Novel Proteins Downstream of Nox1 Activation in VSMC

**Figure 1:**
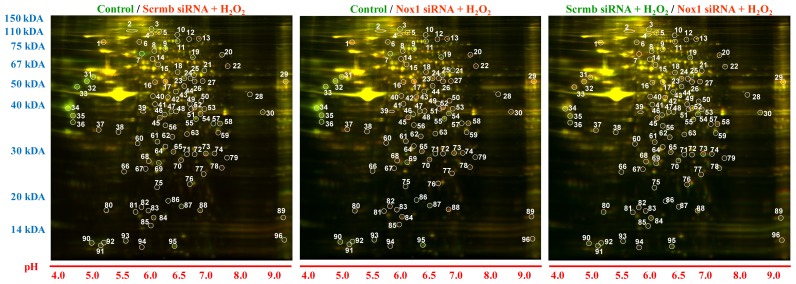
Representative 2D gel images. Vascular smooth muscle cells (VSMC) incubated with vehicle (control) or transfected with Scrmb or Nox1 siRNA and treated with H_2_O_2_ (50 μM, 3 h) were lysed and fluorescently labeled with Cy3, Cy2, or Cy5, respectively and run on a 2D gel (*n* = 1). Images show the comparisons of two fluorescent channels at a time (assigned green and red pseudocolors) as presented in Table 1. Vertical scale indicates the molecular weight ladder; lower horizontal scale denotes pH gradient. The 96 gel spots selected by DeCyder are annotated by white numbers.

To identify proteins upregulated or downregulated by feed-forward ROS-induced Nox1 activation, rat aortic VSMC transfected with scrambled (Scrmb) or Nox1 short interfering RNA (siRNA) were incubated with H_2_O_2_ (50 μM, 3 h) and compared to each other and to vehicle-treated non-transfected VSMC (control). Utilization of fluorescent dyes that complex with all proteins in a sample allows for use of 2D gel technology to compare multiple samples simultaneously. Taking advantage of this technique, each of the three samples were labeled with one particular CyDye (Cy3 for control, Cy2 for Scrmb siRNA + H_2_O_2_, and Cy5 for Nox1 siRNA + H_2_O_2_) and loaded simultaneously onto a 2D gel. For the analysis, two conditions were compared at a time using the DeCyder 2D Differential Analysis Software (version 6.5) as detailed in Table 1. Ninety six gel spots were selected for further analysis based on spot intensity changes and in-gel resolution (Figure 1).

**Figure 2:**
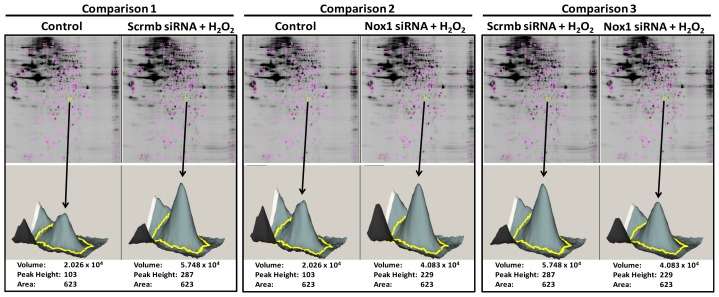
3D view of representative spot intensity. Spot 63 is shown with the DeCyder calculation of fluorescence intensity for each treatment group per comparison. The selected spot is marked by a yellow circle on the 2D gel (*n* = 1).

For each of the identified gel spots, fluorescence intensity from the two wavelengths as well as spot areas were quantified using the DeCyder software. Next, 3D intensity plots were generated and compared between treatment conditions. Figure 2 shows a representative 3D image for spot “63”, which corresponds to ARPC2 (see Table 2).

VSMC proteins either up or downregulated in a Nox1-dependent mechanism were identified if they satisfied the following criteria:

(a) Comparison 1: “Scrmb siRNA + H_2_O_2_”/“control” ≥ 1.3-fold or ≤ 0.7-fold
(b) Comparisons 1 & 2: (“Scrmb siRNA + H_2_O_2_”/“control”) − (“Nox1 siRNA + H_2_O_2_”/“control”) ≥ 0.3-fold or ≤ −0.3-fold
(c) Comparison 3: “Scrmb siRNA + H_2_O_2_”/“Nox1 siRNA + H_2_O_2_” ≥ 0.2 fold. Control connotes vehicle treatment in untransfected VSMC.

The criterion for “c” was selected to reflect a 0.2-fold change in the positive or negative direction (rather than 0.3-fold) to limit the stringency and increase the number of potential candidates of interest.

Using these criteria, 10 spots were selected for protein identification. Of these, 3 spots exhibited an increase in intensity following treatment relative to control, corresponding to protein upregulation. The remaining 7 spots showed a decrease in intensity, which corresponds to protein downregulation in response to H_2_O_2_ (Table 2). The selected spots were analyzed by MS followed by a proteomic *in silico* determination of protein identities within each spot (Table 2).

From the identified proteins, ARPC2 displayed no previous link to Nox isozymes or ROS. Accordingly, we selected this protein as a potential new signaling mediator modulated by Nox1. ARPC2 is 34 kDa protein that together with ARP2, ARP3, ARPC1B, ARPC3, ARPC4, and ARPC5 form the ARP2/3 complex [13]. The ARP2/3 complex is involved in regulation of actin cytoskeleton, functioning as a nucleation site for new actin filament formation and therefore cytoskeletal branching [14]. Previous data reported a link between ARP2/3 complex and cell migration [15–17]. Nevertheless, the role of ARPC2 in potential modulation of cell migration, particularly under oxidative conditions remained unexplored.

### 2.2. Upregulation of ARPC2 Protein Expression in VSMCs via Nox1

**Figure 3:**
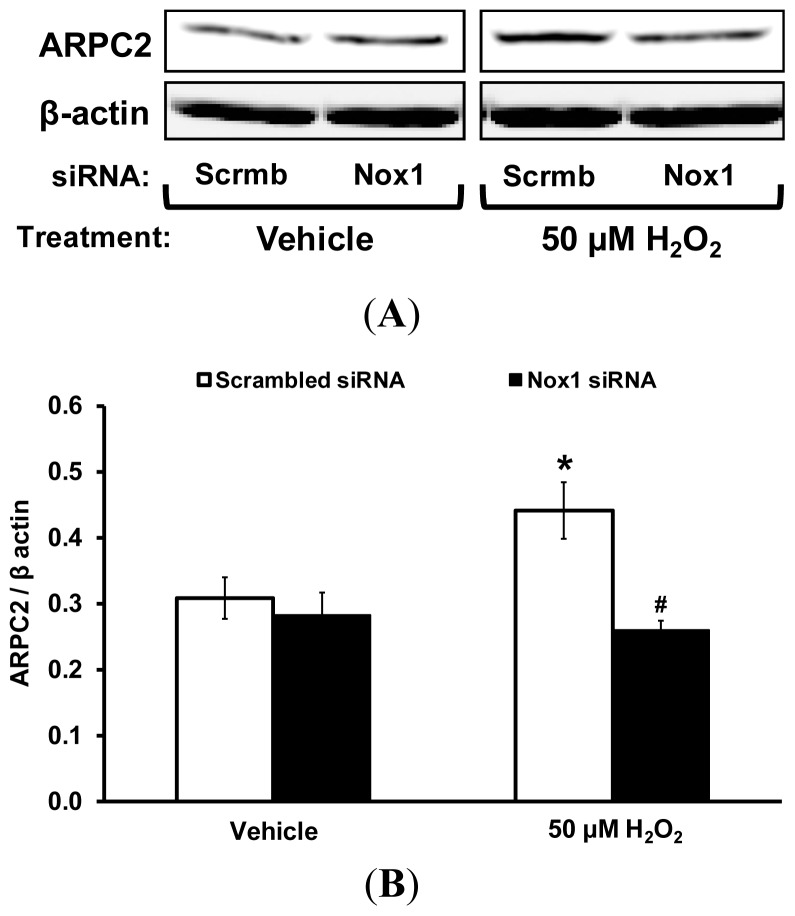
Upregulation of ARPC2 protein expression in VSMCs via Nox1. Nox1 and Scrmb siRNA-treated VSMC were incubated with vehicle or 50 μM H_2_O_2_ for 3 h. VSMC lysates were subjected to Western blot and probed with a polyclonal antibody against ARPC2 (Aviva Systems Biology) and β-actin (Santa Cruz Biotechnology). (**A**) Representative Western blot; (**B**) Bar graphs representing averaged optical density data expressed as a ratio of ARPC2 to β-actin (*n* = 6). Data represent the mean ± SEM. *p < 0.05 *vs*. Scrmb siRNA + vehicle treatment; ^#^*p* < 0.05 *vs*. Scrmb siRNA + H_2_O_2_ treatment.

To verify the reliability of proteomic analysis, ARPC2 protein expression was analyzed by Western blot. VSMC were transfected with Nox1 or Scrmb siRNA and treated with vehicle or 50 μM H_2_O_2_ for 3 h. Nox1 protein expression was previously determined to be suppressed by ~70% using Nox1 siRNA without affecting Nox4 expression [10] (Nox2 and Nox5 are not expressed in rat aortic VSMC [7,10]). As shown in Figure 3, treatment of VSMC with H_2_O_2_ significantly increased ARPC2 expression. Gene silencing of Nox1 using siRNA significantly decreased ARPC2 expression in H_2_O_2_-treated VSMC.

### 2.3. H_2_O_2_ Stimulates VSMC Migration via Nox1

**Figure 4:**
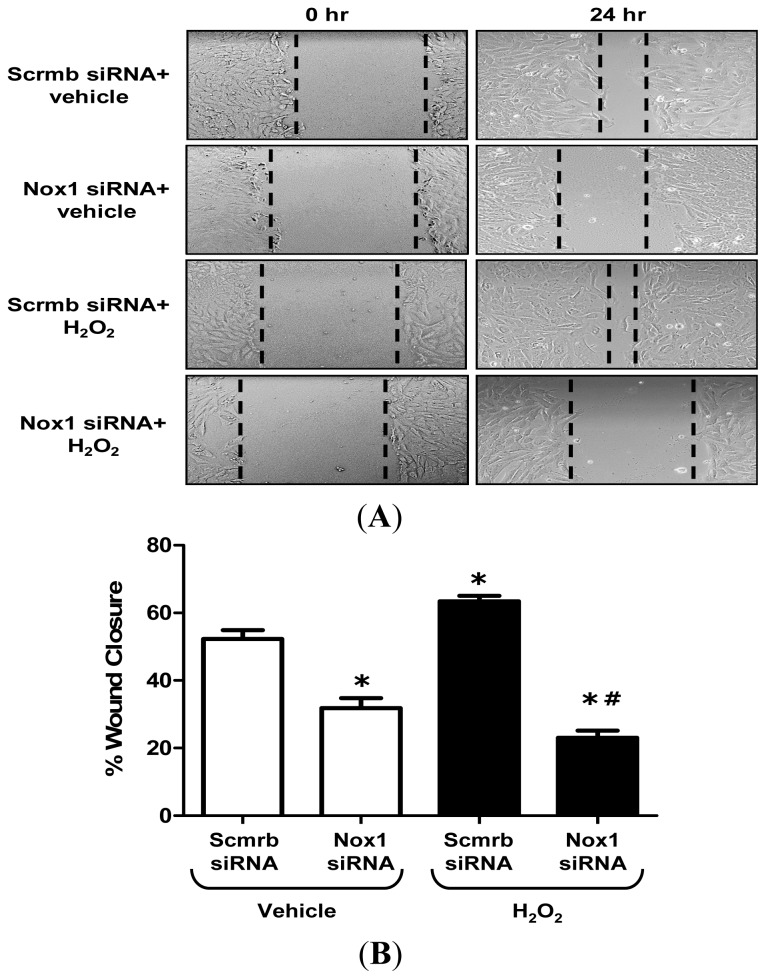
H_2_O_2_ stimulates VSMC migration via Nox1. VSMC were transfected with Scrmb or Nox1 siRNA and treated with vehicle or H_2_O_2_ (50 μM). Cell migration was determined by wounding of VSMC monolayers. Wound area was monitored at 0 and 24 h. (**A**) Representative images taken at 0 and 24 h after wounding (original magnification, ×10). Dashed lines denote the approximate edge of the wound at these time points; (**B**) Quantitative assessment of the data (*n* = 3). Data represent the mean ± SEM. * *p* < 0.05 *vs*. Scrmb siRNA + vehicle treatment; ^#^*p* < 0.05 *vs*. Scrmb siRNA + H_2_O_2_ treatment.

ROS mediate important cellular processes including, but not limited to, migration and proliferation [4,5]. To confirm the role of Nox1 in VSMC migration in our experimental setting, Nox1 and Scrmb siRNA-transfected VSMC were wounded by scratching the cellular monolayer (0 h) and incubated for 24 h with vehicle or H_2_O_2_ (50 μM). As shown in Figure 4, H_2_O_2_ treatment significantly increased migration of Scrmb siRNA-transfected VSMC compared to vehicle treatment. In addition, gene silencing of Nox1 completely abolished the effect of H_2_O_2_ on VSMC migration, demonstrating for the first time that H_2_O_2_ stimulates VSMC migration via Nox1. Taken together with our previous findings [10,18], these data further support the notion of ROS as positive feed-forward regulators of Nox.

**Figure 5:**
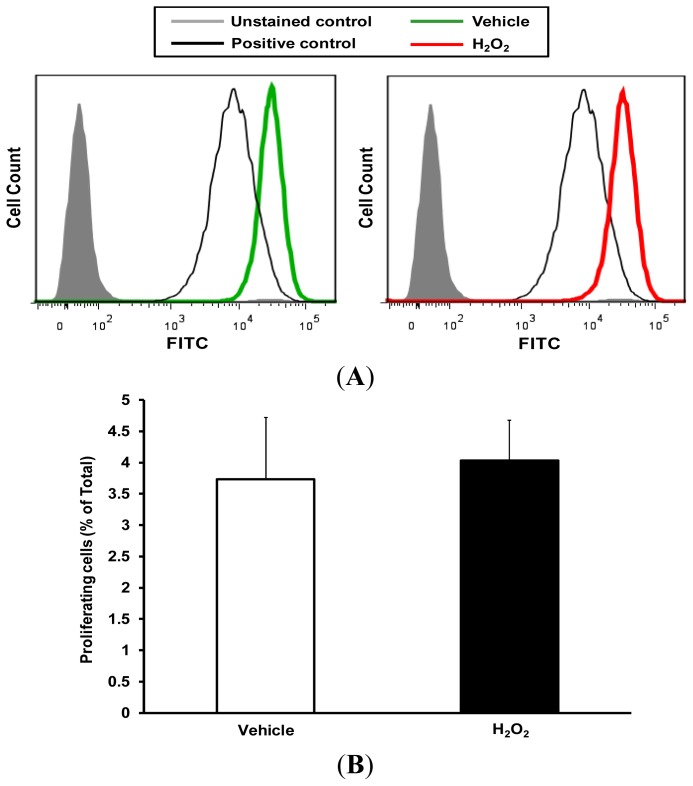
H_2_O_2_ (50 μM) tyreatment for 24 h does not stimulate VSMC proliferation. serum-starved (0.1% FBS, 24 h) VSMC were pretreated with CFSE (1.25 μM, 15 min) and incubated with vehicle or 50 μM H_2_O_2_. Twenty four hours later CFSE fluorescence was evaluated using a BD LSRFortessa™ cell analyzer and flow data were analyzed using FlowJo (version 10). (**A**) Histograms showing CFSE fluorescence intensities in unstained controls (grey fill) and CFSE-stained VSMC treated with vehicle (green tracing) or H_2_O_2_ (red tracing). VSMC incubated for 48 h in the presence of complete growth media (10% fetal bovine serum; FBS) were used as positive controls (black tracing); (**B**) Quantitative assessment of VSMC proliferation (*n* = 3). Data represent the mean ± SEM.

To investigate whether increased cellular proliferation in response to H_2_O_2_ treatment contributes to wound closure, VSMC proliferation was investigated using the carboxyfluorescein diacetate succinimidyl ester (CFSE) proliferation assay. Cells were treated with vehicle or H_2_O_2_ (50 μM) and proliferation was determined by the extent of reduction in CFSE fluorescence as a consequence of dilution into daughter cells at 24 h. Figure 5 demonstrates that H_2_O_2_ treatment did not increase VSMC proliferation following 24 h of treatment (proliferating cells: 3.7 ± 0.9 and 4.0% ± 0.6% of total for vehicle and H_2_O_2_ treatments, respectively).

### 2.4. Gene Silencing of ARPC2 Attenuates VSMC Migration

**Figure 6:**
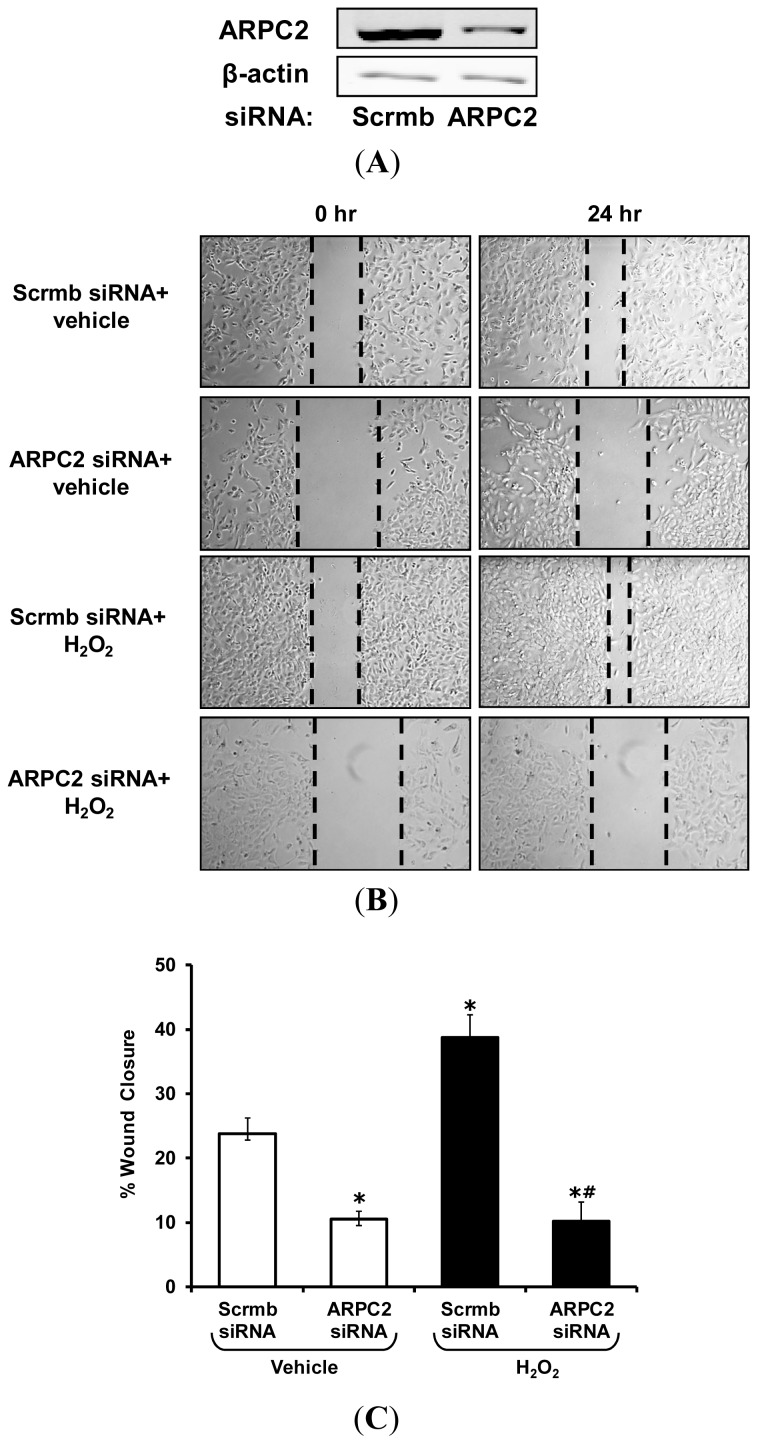
H_2_O_2_ stimulates VSMC migration via ARPC2. ARPC2 and Scrmb siRNA-transfected VSMC were incubated with vehicle or H_2_O_2_ (50 μM) for 24 h. Cell migration was determined by wounding of VSMC monolayers. Images were captured at 0 and 24 h. (**A**) Representative Western blot showing ARPC2 and β-actin expression in Scrmb- and ARPC2-siRNA-treated VSMC. Images are representative of three independent experiments with similar results; (**B**) Representative images of wound healing at 0 and 24 h after scratch are shown (original magnification, ×5). Dashed lines denote the approximate edge of the wound at 0 and 24 h time points; (**C**) Quantitative assessment of VSMC migration (*n* = 6). Data represent the mean ± SEM. * *p* < 0.05 *vs*. Scrmb siRNA + vehicle treatment; ^#^*p* < 0.05 *vs*. Scrmb siRNA + H_2_O_2_ treatment.

To investigate whether ARPC2 plays a role in VSMC migration, we used siRNA to gene silence ARPC2, treated VSMC with vehicle or H_2_O_2_, and measured migration as described in Figure 4. ARPC2 protein expression was suppressed 69% by ARPC2 siRNA compared to Scrmb control (Figure 6A). H_2_O_2_ treatment significantly increased migration of Scrmb siRNA-treated VSMC compared to vehicle treatment (Figure 6B,C). Importantly, gene silencing of ARPC2 abolished the effect of H_2_O_2_ on VSMC migration. These data demonstrate that H_2_O_2_ stimulates VSMC migration via ARPC2, providing a new role for ARPC2 as a downstream modulator of Nox signaling and possibly the effector of Nox1-induced VSMC migration.

### 2.5. Pharmacological Inhibition of p38 MAPK Attenuates H_2_O_2_-Induced ARPC2 Expression

**Figure 7:**
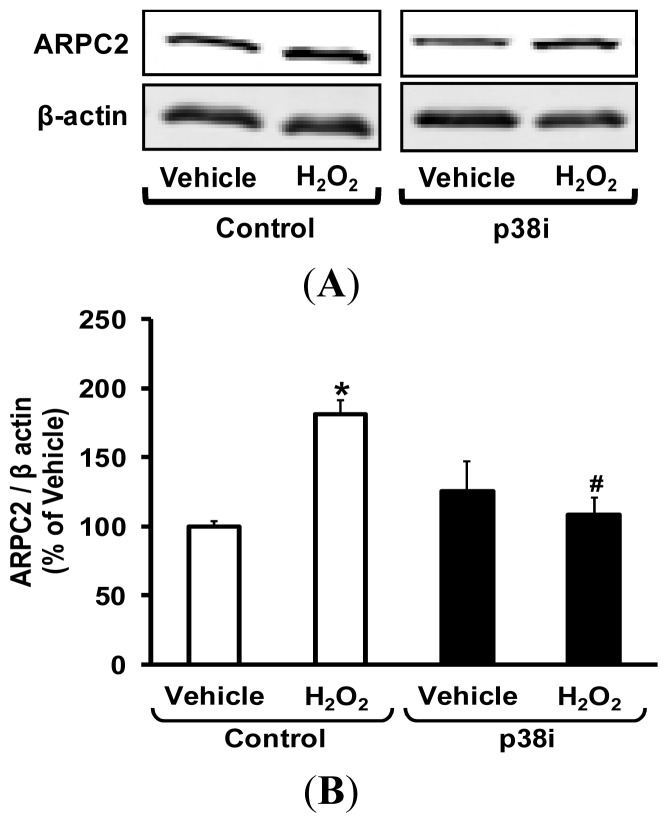
Inhibition of p38 MAPK pathway attenuates H_2_O_2_-induced ARPC2 expression. VSMC were pre-treated with the p38 MAPK inhibitor (p38i) SB203580 (1 μM, 1 h, Millipore), followed by treatment with vehicle or H_2_O_2_ (50 μM) for 3 h. VSMC lysates were subjected to Western blot and probed with polyclonal antibodies against ARPC2 and β-actin. (**A**) Representative Western blot showing ARPC2 and β-actin expression in control- and p38i-treated VSMC incubated with vehicle or H_2_O_2_; (**B**) Bar graphs representing averaged optical density data expressed as a ratio of ARPC2 to β-actin (*n* = 4). Data represent the mean ± SEM. *p < 0.05 *vs*. Control + vehicle treatment. ^#^*p* < 0.05 *vs*. Control + H_2_O_2_ treatment.

Nox enzymes are known to activate p38 mitogen-activated protein kinase (p38 MAPK) [19–21] and an *in vivo* connection between H_2_O_2_ and p38 MAPK was recently demonstrated by our laboratory [18]. In addition, a recent study demonstrated that pharmacological inhibition of p38 MAPK inhibits VSMC migration *in vitro* [22]. Consequently, we investigated the involvement of p38 MAPK as a potential signaling mediator in oxidant-induced Nox1-dependent ARPC2 expression and VSMC migration. Indeed, while an association may be inferred as such [23,24], to date no connection has been demonstrated between p38 MAPK and ARPC2. To test this hypothesis, VSMC were transfected with Scrmb or Nox1 siRNA and treated with H_2_O_2_ (50 μM, 3 h), followed by Western blot for phospho- and total-p38 MAPK kinase. As shown in Supplemental Figure S1, H_2_O_2_ treatment significantly increased the level of phospho-p38 relative to total p38 (indicative of its activation) compared to vehicle treatment. Gene silencing of Nox1 abolished p38 MAPK phosphorylation in H_2_O_2_-treated VSMC. These data indicate that H_2_O_2_ leads to activation of p38 MAPK in a Nox1-dependent mechanism. To investigate whether p38 MAPK is upstream of Nox1-induced ARPC2 expression, VSMC were incubated with the p38 MAPK inhibitor SB203580 (1 μM, 1 h) before H_2_O_2_ treatment. As shown in Figure 7, inhibition of p38 MAPK attenuated H_2_O_2_-induced ARPC2 protein expression, consistent with a role for p38 MAPK activation in this process.

Since p38 MAPK signaling is linked to NF-κB activation [25,26], we further investigated whether inhibition of the NF-κB pathway attenuates ARPC2 expression in response to H_2_O_2_. One strategy used to block the NF-κB pathway is the pharmacological inhibition of IκB kinase (IKK). IKK is responsible for phosphorylating the inhibitory IκB protein, thereby releasing it from NF-κB, allowing for NF-κB activation [27]. VSMC were pre-treated with the IKK inhibitor wedelolactone (60 μM 1 h, Millipore) [27,28], incubated with H_2_O_2_, and investigated for ARPC2 expression. Interestingly, our findings demonstrated that pharmacological inhibition of IKK does not inhibit H_2_O_2_-induced ARPC2 expression (data not shown). These data suggest that a transcription factor other than NF-κB is responsible for oxidant-induced Nox1- and p38 MAPK-mediated ARPC2 expression. Our data are also consistent with the literature demonstrating that higher concentrations of H_2_O_2_ than were used in the present study may be required for NF-κB activation [29].

Further studies are required to identify this transcription factor in VSMC. It is also possible that post-translational and post-transcriptional modifications of ARPC2 regulated by the Nox1-p38 MAPK pathway may play a role. For example, attenuated ubiquitination or increased mRNA stability may lead to increased ARPC2 protein levels independent of *de novo* mRNA expression. Indeed, p38 MAPK has been shown to increase mRNA stability in vascular cells [30]. Taken together, the data presented herein suggest a signaling pathway whereby Nox1-derived ROS lead to increased ARPC2 protein expression via p38 MAPK kinase phosphorylation. The activation of this pathway leads to increased VSMC migration *in vitro*, suggesting a potentially important role of this signaling cascade in vascular hyperplasia and related pathologies *in vivo*.

## 3. Experimental Section

### 3.1. Cell Culture

Rat aortic vascular smooth muscle cells (VSMC; Lonza, Walkersville, MD, USA) were grown in Dulbecco’s Modified Eagle’s medium (Mediatech, Inc. Manassas, VA, USA) containing 10% heat-inactivated fetal bovine serum (FBS), 100 units/mL penicillin, and 100 μg/mL streptomycin. Cells were used at passages 3–10 [31].

### 3.2. Gene Silencing

VSMC were grown to 30%–50% confluence and transfected with siRNA against Nox1 or ARPC2 (Stealth RNAi™, Invitrogen, Carlsbad, CA, USA) using the transfection reagent Lipofectamine 2000 (Invitrogen, Carlsbad, CA, USA) according to the manufacturer’s protocol. To control for possible non-specific effects of siRNA, Stealth RNAi™ siRNA negative controls were applied. 48 h after transfection VSMC were serum starved (0.1% FBS; 24 h), treated with vehicle or H_2_O_2_ (50 μM, 3 h), and assayed. Knockdown of Nox1 was confirmed previously [10]. ARPC2 knockdown was confirmed by Western blot.

### 3.3. Western Blot

Western blot was used to assess ARPC2 expression and p38 MAPK phosphorylation and expression in response to experimental manipulation. Seventy two hours after transfection, VSMC lysates were subjected to SDS-PAGE/Western blot with a polyclonal antibody against ARPC2 (Aviva Systems Biology, San Diego, CA, USA), total p38 MAPK (Cell Signaling) and β-actin (Santa Cruz Biotechnology, Dallas, TX, USA) and monoclonal antibody against phospho-p38 MAPK (Cell Signaling). Membranes were then incubated with the appropriate secondary antibodies (Li-Cor Biotechnology, Lincoln, NE, USA). Blots were scanned using the Odyssey Infrared Imaging System (Li-Cor Biotechnology) and blot density was quantified using ImageJ software (http://rsbweb.nih.gov/ij/).

### 3.4. Proteomic Analysis Using Two-Dimensional Differential In-Gel Electrophoresis (2D-DIGE) Combined with Mass Spectroscopy (MS)

#### 3.4.1. 2D-DIGE

2D-DIGE and protein identification by mass spectroscopy were performed by Applied Biomics, Inc. (Hayward, CA, USA). Cultured VSMC were transfected with Scrmb or Nox1 siRNA (Invitrogen), treated with vehicle or 50 μM H_2_O_2_ for 3 h, and 5 mg of pelleted cells were lysed in 100 μL of 2D cell lysis buffer (30 mM Tris-HCl, pH 8.8, containing 7 M urea, 2 M thiourea, and 4% CHAPS). Protein concentration was measured using the Bio-Rad method (Hercules, CA, USA). 30 μg of protein were mixed with 1.0 μL of diluted CyDye (GE Healthcare, Uppsala, Sweden) and kept in the dark on ice for 30 min. The labeling reaction was stopped by adding 1.0 μL of 10 mM lysine to each sample and incubating in the dark on ice for an additional 15 min. The labeled samples were then mixed together. 2× 2D gel sample buffer (8 M urea, 4% CHAPS, 20 mg/mL DTT, 2% pharmalytes, and trace amount of bromophenol blue), and 100 μL destreak solution and rehydration buffer (7 M urea, 2 M thiourea, 4% CHAPS, 20 mg/mL DTT, 1% pharmalytes, and a trace amount of bromophenol blue) were added to the labeling mix to bring the total volume to 250 μL. The samples were mixed and centrifuged before loading into the IPG strip holder for sample loading. After loading the labeled samples, isoelectric focusing (IEF; pH 3–10 Linear) was run following the protocol provided by GE Healthcare. The immobilized pH gradient (IPG) strips were incubated in freshly made equilibration buffer-1 (50 mM Tris-HCl, pH 8.8, containing 6 M urea, 30% glycerol, 2% SDS, a trace amount of bromophenol blue, and 10 mg/mL DTT) for 15 min with gentle shaking. The strips were rinsed in freshly made equilibration buffer- (50 mM Tris-HCl, pH 8.8, containing 6 M urea, 30% glycerol, 2% SDS, trace amount of bromophenol blue, and 45 mg/mL iodoacetamide) for 10 min with gentle shaking. Next the IPG strips were rinsed in the SDS-gel running buffer before transfer onto 12% SDS-gels. The SDS-gel was run at 15 °C until the dye front ran out of the gels. Gel images were scanned immediately following the SDS-PAGE using Typhoon TRIO (GE Healthcare). The scanned images were analyzed by Image Quant software (version 6.0, GE Healthcare), followed by in-gel analysis using DeCyder software version 6.5 (GE Healthcare). The fold change of protein expression levels was obtained from in-gel DeCyder analysis.

#### 3.4.2. Mass Spectrometry

The spots of interest were “cherry-picked” by Ettan Spot Picker (GE Healthcare) based on the in-gel analysis and spot selection design by DeCyder software. Gel spots were washed a few times then digested in-gel with modified porcine trypsin protease (Promega, Madison, WI, USA). The digested tryptic peptides were desalted using a Zip-tip C18 (Millipore, Billerica, MA, USA). Peptides were eluted from the Zip-tip with 0.5 μL of matrix solution (α-cyano-4-hydroxycinnamic acid 5 mg/mL in 50% acetonitrile, 0.1% trifluoroacetic acid, 25 mM ammonium bicarbonate) and spotted on a MALDI plate. MALDI-TOF MS and TOF/TOF tandem MS/MS were performed on AB SCIEX TOF/TOF™ 5800 System (AB SCIEX, Framingham, MA, USA). MALDI-TOF mass spectra were acquired in reflectron positive ion mode, averaging 4000 laser shots per spectrum. TOF/TOF tandem MS fragmentation spectra were acquired for each sample, averaging 4000 laser shots per fragmentation spectrum on each of the 7–10 most abundant ions present in each sample (excluding trypsin autolytic peptides and other known background ions). Both of the resulting peptide mass and the associated fragmentation spectra were submitted to GPS Explorer workstation equipped with MASCOT search engine (Matrix Science, Boston, MA, USA) to search the database of National Center for Biotechnology Information, non-redundant (NCBI-nr). Searches were performed using the following criteria in the search parameters: (1) no constraint of protein molecular weight or isoelectric point; (2) allowing for variable carbamidomethylation of cysteine and oxidation of methionine residues, and (3) allowing for one missed cleavage. Candidates with either protein score C.I.% or Ion C.I.% greater than 95 were considered significant.

### 3.5. Wound Migration “Scratch” Assay

VSMC monolayers were serum starved (0.1% FBS; 24 h) and scratched/wounded using a sterile 200 μL pipette tip (BD Biosciences, San Diego, CA, USA). Each experiment was run in triplicates. For each replicate 6 images were captured for assessing wound area. Photos were taken immediately after wounding (0 h) and at 24 h post-wounding using a Zeiss Axiovert 40 CFL microscope. To quantify migration, wound area was calculated using ImageJ software (http://rsbweb.nih.gov/ij/) at 0 and 24 h time points, and expressed as % wound closure for each treatment.

### 3.6. Carboxyfluorescein Succinimidyl Ester (CFSE) Quantification

VSMC proliferation was assessed using the CellTrace™ CFSE Cell Proliferation Kit according to the manufacturer’s instructions (Invitrogen). VSMC were seeded into 6-well tissue culture dishes, serum starved (0.1% FBS) for 24 h, and incubated with 1.25 μM CFSE for 15 min in phosphate-buffered saline (PBS). Cells were washed with PBS and incubated with vehicle or H_2_O_2_ (50 μM) for 24 h. VSMC incubated for 48 h in the presence of complete growth media (10% FBS) were used as positive controls. CFSE fluorescence was evaluated using a BD LSRFortessa™ cell analyzer and flow data were analyzed using FlowJo (version 10).

### 3.7. Statistical Analysis

All results are expressed as mean ± SEM. Significance of the differences were assessed by Student’s *t*-test, one- or two-way ANOVA followed by a Bonferroni post-hoc test as appropriate for the particular experiment and treatment groups. A value of *p* < 0.05 was considered to be statistically significant.

## 4. Conclusions

In this manuscript, we identify ARPC2 as a new downstream target of Nox1 activation and demonstrate the importance of Nox1/ARPC2 signaling axis in VSMC migration. The data presented herein also support a role for p38 MAPK as an upstream mediator of Nox1-induced ARPC2 expression and VSMC migration. This previously unreported signaling pathway sheds important light on the pathological role of Nox/ROS in VSMC and is expected to open a new area of investigation into the role of ARPC2 in vascular disease. The current work also introduces a potential therapeutic target downstream of Nox1 that may offer alternative strategies for disruption of pro-migratory pathways under conditions of oxidative stress.

## Figures and Tables

**Table 1:** Comparisons among treatment groups conducted by the DeCyder software.

Comparison	Treatment conditions	
1	Control	Scrmb siRNA + H_2_O_2_
2	Control	Nox1 siRNA + H_2_O_2_
3	Scrmb siRNA + H_2_O_2_	Nox1 siRNA + H_2_O_2_

**Table 2:** List of VSMC proteins up or downregulated by H_2_O_2_ treatment in a Nox1-dependent manner.

Spot number	Protein name	Up/Down-regulation	Accession No.	Protein Score	Molecular weight (Da)	Protein Score C. I.%/Total Ion C. I.%	Major function
2	Fibronectin 1, isoform CRA_d	↓	gi|149015981	411	262,586	100/100	Cell adhesion and differentiation
4	Heterogeneous nuclear ribonucleoprotein U	↑	gi|148747541	601	87,678	100/100	Regulation of mRNA metabolism and transport
7	Fibronectin 3	↓	gi|204158	111	74,876	100/100	Wound healing, cell adhesion and differentation
32	Vimentin, isoform CRA_b	↓	gi|149021114	1,250	53,668	100/100	Type III intermediate filament, maintains cellular integrity
33	Vimentin	↓	gi|14389299	1350	53,700	100/100	Type III intermediate filament, maintains cellular integrity
57	Insulin-like growth factor (IGF)-binding protein 2 precursor	↓	gi|148747421	584	32,833	100/100	Regulates bioavailability of IGF
59	36 kda voltage dependent anion channel	↑	gi|299036	355	31,700	100/100	Voltage-dependent anion channel protein
63	ARPC2	↑	gi|205686193	823	34,369	100/100	Regulation of actin cytoskeleton
86	Cofilin-1	↓	gi|8393101	148	18,520	100/100	Regulation of actin cytoskeleton dynamics
87	Destrin	↓	gi|62665569	241	18,507	100/100	Actin-depolymerizing protein
